# Analysis of the Effect of Training Driving on Electromyographic Parameters in Trained Karting Drivers: A Study of Fatigue and Its Relationship with Training Practice

**DOI:** 10.3390/jfmk10020190

**Published:** 2025-05-26

**Authors:** Aleksander Zarębski, Marcel Słomiński, Małgorzata Smoter, Rafał Studnicki

**Affiliations:** 1 Student Scientific Circle of Orthopaedic Manual Therapy, Medical University of Gdańsk, 7 Dębinki Street, 80-211 Gdańsk, Poland; 2 Department of Basic Physiotherapy, Gdańsk University of Physical Education and Sport, 1 Kazimierza Górskiego Street, 80-336 Gdańsk, Poland; malgorzata.smoter@awf.gda.pl; 3 Department of Physiotherapy, Medical University of Gdańsk, 7 Dębinki Street, 80-211 Gdańsk, Poland

**Keywords:** electromyography, motor control, fatigue, go-karting

## Abstract

**Objectives:** The present study aimed to investigate changes in forearm muscle activity associated with short-term go-kart driving (680 m) and its potential effect on muscle activation patterns. **Methods**: Eleven male karting league drivers (mean age: 23.18 ± 1.40 years; body mass: 83.27 ± 10.98 kg; height: 182.73 ± 5.66 cm) volunteered to participate. Electromyographic (EMG) activity was recorded from four muscles: extensor carpi radialis (ECR), extensor carpi ulnaris (ECU), flexor carpi radialis (FCR), and flexor carpi ulnaris (FCU). Baseline EMG was measured before the intervention, followed by two consecutive kart-driving sessions on a 680 m closed track. Post-exercise EMG data were then collected. A repeated-measures analysis of covariance (ANCOVA) was used to analyze the effects of time (pre vs. post) while controlling for cumulative race time as a covariate. **Results**: A significant time effect with cumulative time as a covariate was observed, particularly in the ECR and ECU muscles on both the left and right sides. Notable findings include increases in maximum and mean activity of the left and right ECR (e.g., ECR right max: F = 51.57; *p* < 0.001; η^2^ = 0.851) and ECU (e.g., ECU right max: F = 36.170; *p* < 0.001; η^2^ = 0.801). Additionally, a significant increase was found in the maximum activation of the left FCR (F = 11.019; *p* = 0.009; η^2^ = 0.550, which remained significant after controlling for total driving time. This heightened activation likely reflects an acute neuromuscular fatigue response to the demands of kart steering, rather than a long-term adaptation. **Conclusions**: The findings suggest that even short bouts of kart driving can induce measurable changes in neuromuscular activation of the forearm muscles, particularly in those involved in grip control and steering stability. This highlights the physical demands of karting and its potential impact on the upper limb muscle conditioning.

## 1. Introduction

Go-karting, as a motorsport discipline, requires not only precise driving skills but also significant physical effort from competitors. During races, drivers are subjected to intense overloads, vibrations, and repetitive movements, all of which can lead to various types of injuries. With the increasing popularity of go-karting, both as a recreational activity and a competitive sport, more individuals are exposed to varying degrees of physical demands and injury risks. Recreational karting, often practiced in indoor or rental facilities, typically involves lower speeds and shorter sessions but still presents a considerable risk, particularly due to inexperience or lack of proper instruction. In contrast, competitive karting involves higher speeds, more intensive training, and greater cumulative physical stress, which may increase the likelihood of overuse injuries, particularly in the upper limbs. A study conducted in the Netherlands found that approximately 600 people annually require medical attention due to injuries sustained while driving karts, confirming the high risk of injury associated with this activity [1]. Additionally, go-karting plays a key role in the careers of professional racing drivers, and it is the most commonly chosen form of motorsport for beginners [2].

Research on go-kart racing and muscle fatigue highlights the potential injury risks linked to physical exertion. Studies examining the development of fatigue during extended driving sessions, using tools such as postural force platforms and electromyography (EMG) signals, have shown that factors like exposure time, track conditions, and even gender influence muscle fatigue [3,4]. One critical factor in maintaining vehicle control, handgrip strength, may be compromised during prolonged driving sessions [5]. Electromyography (EMG) analysis of the brachioradialis and biceps brachii muscles is commonly employed to assess muscle fatigue during simulated driving. Various signal processing techniques are used to extract meaningful features from EMG data. In the time domain, parameters such as mean, variance, and root mean square (RMS) provide insights into the amplitude of the signal, which reflects the level of muscle activation. Increases in EMG amplitude are often associated with higher motor unit recruitment in response to fatigue. In the frequency domain, analyses such as Power Spectral Density (PSD) and Fast Fourier Transform (FFT) allow researchers to monitor shifts in spectral content, particularly median or peak frequency. A decrease in these frequencies typically indicates a reduction in muscle fiber conduction velocity, a well-established marker of neuromuscular fatigue. For example, EMG analysis of the brachioradialis muscle during simulated driving revealed changes in signal amplitude and variance, along with shifts in the peak frequency of the power spectral density, all of which are indicative of progressive muscle fatigue [6]. Furthermore, advanced classification methods, including Naïve Bayes and linear discriminant analysis, have been used to distinguish different levels of fatigue based on these EMG features [6,7]. During competitions, drivers race lightweight, highly maneuverable vehicles that generate significant overloads, particularly in turns. Every movement of the steering wheel has a direct impact on the kart’s trajectory, requiring substantial involvement of the forearm muscles. Centrifugal forces, vibrations, and repetitive movements intensify the work of these muscles, which, over time may lead to overloading of the elbow joint, resulting in injuries such as “tennis elbow.” This injury occurs due to excessive strain at the common attachment site of the forearm extensors on the lateral epicondyle of the humerus [8].

Despite the high physical demands and risks associated with go-karting, research on karting-related injuries remains limited [9]. Studies on injury mechanisms in go-karting reveal that the most common injuries involve the hands and wrists (42.1%), followed by chest and elbow injuries (13.1%) [10]. Broader motorsport studies report a notable prevalence of acute injuries, with forearm injuries representing about 7% of cases [11]. Fatigue in the forearm muscles has been identified as a critical factor that can impair performance and increase the risk of musculoskeletal disorders, particularly among motorcycle riders [12]. Prolonged driving sessions have been shown to negatively affect handgrip force, a crucial component in maintaining control over the vehicle and counteracting the centrifugal forces experienced during high-speed turns [5].

Research has established a significant link between muscular fatigue and reduced grip strength. Hawkes et al. [13] reported a decline in grip strength following exhaustive shoulder exercise, with incomplete recovery of EMG fatigue indices despite restoration of strength levels, suggesting sustained neuromuscular fatigue. Similarly, Fernandes et al. [14] (20 observed a progressive decline in handgrip strength across multiple trials, indicating the cumulative effects of peripheral fatigue. Souza et al. [15] found that fatigue in the wrist extensor muscles caused reductions in both handgrip and lateral pinch strength, as confirmed via surface EMG analysis. Emge et al. [16] further demonstrated that muscular fatigue disrupts grip force modulation and load force coupling during manipulation tasks, which may increase the risk of dropping objects or loss of control, although task accuracy may be preserved under moderate fatigue. These findings highlight the functional consequences of muscle fatigue on grip efficiency and suggest that even mild neuromuscular fatigue can compromise performance and increase injury risk during motorsport activity. To better understand the neuromuscular demands of racing, intermittent fatigue protocols have been employed to simulate real-world driving conditions, revealing that riders exhibit significantly higher activation of the carpi radialis muscle compared to non-riders [17]. Furthermore, the development of force-time curve models has enabled researchers to characterize and predict fatigue patterns in forearm muscles, demonstrating variability in how different individuals experience and respond to racing-induced muscle fatigue [12]. Taken together, these findings underscore the importance of further investigating both the acute and chronic impacts of go-karting on upper limb function, particularly as it relates to performance, safety, and injury prevention within motorsport contexts.

Considering the above-mentioned, this study aims to analyze changes in muscle activity during go-kart driving and their effects on muscle activation in adult drivers racing on a closed go-kart track.

## 2. Materials and Methods

The Independent Bioethics Committee for Scientific Research at the Medical University of Gdańsk approved this study on 12 December 2024 (Resolution No. KB/519/2024). Additionally, this study was registered in Clinical Trials: NCT06826300. This study followed the ethical guidelines set forth in the Declaration of Helsinki.

Participants were thoroughly informed about this study, including a simplified overview of the protocol. They provided written informed consent prior to participation, acknowledging their voluntary involvement and their right to withdraw from this study at any time without any consequences.

### 2.1. Study Design

This study employed a non-randomized, uncontrolled design and involved 11 male karting league drivers with a mean age of 23.18 ± 1.40 years, body mass of 83.27 ± 10.98 kg, and height of 182.73 ± 5.66 cm. EMG measurements were taken at baseline and after two consecutive training drives. EMG measurements were taken from the extensor carpi radialis and extensor carpi ulnaris muscles and the flexor carpi radialis and flexor carpi ulnaris muscles.

### 2.2. Setting

The evaluation and experimental intervention took place at the E1GOKART Gdańsk karting track in late 2024. The initial evaluation was conducted on the training day, immediately after the athletes’ warm-up and just before the start of the training. The second evaluation was conducted five minutes after the athletes had performed two consecutive training runs of 680 m (average race time of 58.38 ± 4.96 s). Each volunteer was individually evaluated in the afternoon (around 16:00) after 48 h of rest. A convenience sampling method was used, inviting experienced drivers in the karting league. Recruitment was conducted via club announcements and social media posts.

### 2.3. Participants

The sample size for this study was determined using G*Power (version 3.1.9.6, Universität Düsseldorf, Düsseldorf, Germany), with calculations based on an ANOVA repeated measures within–between interaction. Assuming a medium effect size of 0.5, a power of 0.85, two groups, and three measurements, the recommended sample size was 11 participants.

To be included in this study, participants had to meet the following criteria: (i) be actively training as a licensed driver competing in a karting league, (ii) be in good health with no recent history of injury or illness; (iii) be at least 18 years of age; and (iv) engage in all phases of this study. Exclusion criteria were (i) having sustained an injury or illness during this study; (ii) having had an upper extremity injury (shoulder, elbow, or wrist) in the previous six months, pain in these joints, hypermobility of a lower extremity joint, or any neurological or connective tissue disease.

### 2.4. Procedures

**Figure 1:**
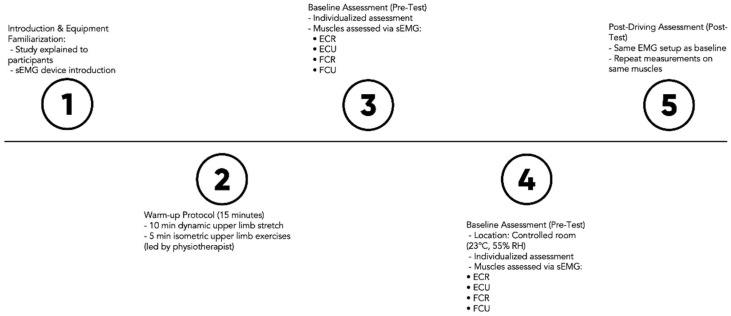
Sequential phases of the implemented protocol. sEMG: surface electromyography; ECR: extensor carpi radialis; ECU: extensor carpi ulnaris; FCR: flexor carpi radialis; FCU: flexor carpi ulnaris.

Muscle fatigue assessment was performed on both limbs. After completing a standardized, investigator-led warm-up protocol—including 10 min of upper extremity dynamic stretching and 5 min of upper extremity isometric exercise—participants completed baseline assessments (Figure 1). All those exercises were administered by a physiotherapist responsible for a group of drivers, and they represent standard routines typically performed prior to driving. These assessments took place on the same track in a dedicated room equipped with EMG instruments, maintained at 23 °C and 55% relative humidity. To minimize athlete disturbance, assessments were conducted individually. Assessments were conducted by two investigators: one specialized in EMG instruments and the other in physical performance assessments. The following diagram depicts the sequential phases of the implemented protocol.

During the initial assessment (before training), participants were given an introduction to this study and familiarized with the equipment. sEMG activity was measured in selected muscles: the extensor carpi radialis (ECR) and ulnaris (ECU) muscles, and the flexor carpi radialis (FCR) and ulnaris (FCU) muscles. Each muscle was assessed three times during the targeting phase [18]. The electrodes were left on the riders throughout the ride and did not change their position.

### 2.5. Measurements

Surface EMG data were captured from the extensor carpi radialis (ECR), extensor carpi ulnaris (ECU), flexor carpi radialis (FCR), and flexor carpi ulnaris (FCU) muscles during the maximum voluntary isometric contraction phase. For each muscle, a 3-s window of EMG activity was recorded and used for subsequent analysis. The SEMG data were gathered and differentially amplified with a gain of 500 using TeleMyo DTS (Noraxon, Scottsdale, AZ, USA) along with Ag/AgCl 1 cm^2^ surface electrodes (Sorimex, Toruń, Poland). Band-pass filtering was applied to the SEMG signals (15–500 Hz), and they were sampled at 1500 Hz (16-bit resolution) using an analog-to-digital converter. Subsequently, the SEMG data were archived and processed further using MyoResearch 2.8 software (Noraxon). Electrode placement and skin preparation, including shaving, abrasion, and cleaning with alcohol, adhered to the SENIAM recommendations. Signal processing involved full rectification and smoothing using the root mean square (EMGRMS) method with a 300-millisecond moving time window. The following SEMG outcomes were analyzed: the mean and maximal amplitude of EMGRMS (in µV) and the median frequency of the raw SEMG signal power spectrum (EMGMED, in Hz).

### 2.6. Statistical Procedures

A preliminary analysis was conducted to assess the assumptions of normality and homogeneity using the Kolmogorov–Smirnov and Levene’s tests, respectively. Results confirmed that both assumptions were met (*p* > 0.05 for normality and homogeneity). To account for the influence of baseline values on the magnitude of post-intervention differences between groups, a repeated-measures ANCOVA was performed, including cumulative race time (the total time recorded across both speed tests) as a covariate. Effect sizes were estimated using partial eta squared (η_p^2). Pairwise comparisons were carried out using Bonferroni adjustment. All statistical analyses were performed using IBM SPSS Statistics for Windows, Version 29.0.2.0 (Armonk, NY, USA: IBM Corp.), with the significance level set at *p* > 0.05.

## 3. Results

A repeated-measures analysis of covariance (ANCOVA), using accumulated race time as a covariate, revealed significant interactions between time (pre- and post-test) and muscle activity for several forearm muscles. Specifically, significant interactions were observed for the maximum and mean activity of the extensor carpi radialis (ECR) on both the left (ECR left max: F = 36.013; *p* < 0.001; $\eta_{p}^{2}$ = 0.80; ECR left mean: F = 6.426; *p* = 0.032; $\eta_{p}^{2}$ = 0.417) and right sides (ECR right max: F = 51.57; *p* < 0.001; $\eta_{p}^{2}$ = 0.851; ECR right mean: F = 44.957; *p* < 0.001; $\eta_{p}^{2}$ = 0.833).

Similarly, the activity of the extensor carpi ulnaris (ECU) also demonstrated significant interactions for both maximum and mean values on both sides (ECU left max: F = 5.55; *p* = 0.043; $\eta_{p}^{2}$ = 0.382; ECU left mean: F = 5.30; *p* = 0.047; $\eta_{p}^{2}$ = 0.371; ECU right max: F = 36.170; *p* < 0.001; $\eta_{p}^{2}$ = 0.801; ECU right mean: F = 30.871; *p* < 0.001; $\eta_{p}^{2}$ = 0.774). Additionally, a significant interaction was found for the maximum activity of the flexor carpi radialis left (FCR left max: F = 11.019; *p* = 0.009; $\eta_{p}^{2}$ = 0.550).

Table 1 presents the descriptive statistics of muscle activity (mean ± standard deviation) at pre-test and post-test time points, the percentage difference, and *p*-values from paired *t*-tests. While the ANCOVA revealed significant interactions, the analysis of percentage differences and paired *t*-tests (which did not control for total race time) showed trends of change in muscle activity following the training sessions. For instance, a statistically significant increase in maximum activity was observed for the ECU right (+2.63%, *p* = 0.021) and FCU right (+2.03%, *p* = 0.020) in the paired *t*-tests. However, for other muscles, the differences between the pre-test and post-test measurements did not reach statistical significance in this univariate analysis.

## 4. Discussion

This study aimed to analyze changes in muscle activity during kart driving and its potential impact on muscle activation in adult kart racing drivers competing on a closed track. The primary finding of this study was the significant influence of these training sessions on the activity of several forearm muscles, even after controlling for the accumulated race time. Specifically, the repeated-measures ANCOVA revealed significant interactions between time (pre- and post-test) and muscle activity for both the maximum and mean activation of the extensor carpi radialis (ECR) and extensor carpi ulnaris (ECU) on both the left and right sides, as well as for the maximum activation of the flexor carpi radialis (FCR) on the left side. These results suggest that even a brief period of kart driving can induce notable alterations in the neuromuscular activation patterns of the forearm muscles in adult racers.

The results of the present study are consistent with previous literature, which indicates that fatigue significantly affects forearm muscle activity and performance in motorsports. In go-karting, a 45-min session was shown to reduce handgrip strength, compromising vehicle control [5]. Similarly, studies on motorcycle riding have reported fatigue in the extensor carpi radialis (ECR), with increased ECR activation potentially serving as a compensatory strategy to improve braking precision [17]. These findings align with our own results; both the maximal and mean EMGRMS values for the right ECR, as well as the mean value for the left ECR, showed a significant increase from pre- to post-test, indicating heightened muscle activation. Interestingly, we also observed an increase in median frequency in the right hand (*p* = 0.018). While this may suggest reduced muscle fatigue or improved neuromuscular efficiency [15,16], it could also reflect an adaptive response through altered motor unit recruitment strategies [15]. Given the complexity of interpreting spectral parameters like median frequency, further investigation is needed to clarify their relationship with functional fatigue in kart racing contexts.

The observed trends to an increase in ECR (mean and max right, and max left) and ECU (mean and max of both sides) activity on both sides likely reflect the drivers’ adaptation to the steering demands of karting. In fact, drivers quickly update their internal model of steering dynamics at the sensorimotor level, with minimal subsequent behavioural adaptation [19]. The constant micro-adjustments required to maintain control on a closed track, particularly during cornering, would necessitate enhanced activation of these wrist extensors for stabilization and precise steering inputs [20]. Similarly, the increased maximum activation of the left FCR could be attributed to the counter-steering actions and grip adjustments frequently employed during left-hand turns, which are common on most racing circuits. Counter-steering is a crucial technique in motorcycle and bicycle steering, where the rider initially steers in the opposite direction of the intended turn [21]. This counterintuitive action is necessary for initiating turns and can be executed by explicitly turning the handlebars or shifting body weight. While karts rely on direct steering input via a steering wheel (rather than leaning), drivers still perform continuous fine-tuned corrections, especially in rapid succession during cornering. These micro-adjustments may mimic some neuromuscular patterns seen in counter-steering, particularly in the increased recruitment of wrist flexors and extensors required to stabilize and redirect the steering column under high-load conditions. Studies have highlighted the importance of upper-limb control and forearm endurance during repeated steering adjustments in motorsports [5,22,23]. Therefore, the increased activation of the left FCR observed in our study could reflect the neuromuscular demand associated with these corrective steering maneuvers, especially during left-hand turns, which are more frequent on many closed circuits. Future research could benefit from direct analysis of steering mechanics (e.g., using telemetry or inertial measurement units) to correlate specific movement patterns with localized muscle activation.

The significant changes in muscle activation patterns suggest the potential for corresponding adaptations in muscle strength with extended training periods. Increased recruitment and potentially altered coordination of the ECR, ECU, and FCR may lead to enhanced isometric and dynamic strength in these muscle groups, which are essential for maintaining steering control and resisting fatigue during prolonged races. Isometric strength training, in particular, has been shown to improve joint-specific strength and sports-related dynamic performance, while inducing less fatigue compared to dynamic training [24]. These neuromuscular adaptations may also contribute to improved steering precision and faster reaction times, ultimately enhancing overall driving performance. For instance, a mathematical model incorporating neuromuscular dynamics demonstrates that reflex actions and muscle cocontraction improve steering angle control and path-following accuracy [25]. Studies comparing motorcycle riders and car drivers emphasize the influence of neuromuscular dynamics on driver–vehicle system stability [26]. Real-world driving experiments reveal that drivers adapt the neuromuscular admittance of their arms during cornering, showing increased stiffness compared to straight-line driving [27].

The localized changes in forearm muscle activity observed in this study align with findings in other precision motor control activities, such as motocross or competitive cycling, where maintaining grip and making fine adjustments under dynamic conditions are critical. For instance, Ref. [28] demonstrated that surface-induced loads transmitted through handlebars during cycling significantly increase activation of forearm flexor and extensor muscles, particularly on uneven terrain, highlighting neuromuscular adaptations for dynamic grip control demands. Similarly, Ref. [29] reported significant neuromuscular fatigue in the forearm muscles of motorcycle riders after submaximal intermittent contractions, emphasizing the critical role of grip endurance and fine motor adjustments during high-speed, vibration-intensive activities. However, the unique combination of sustained isometric contractions and rapid, forceful adjustments inherent to kart steering likely elicits a distinct neuromuscular adaptation profile compared to sports with more cyclical or ballistic movements. Kaufmann et al. (2024) [30] discuss how high-demand motor tasks, such as those found in precision sports, require refined neuromuscular coordination and specific motor control strategies.

Nevertheless, although statistically significant increases were observed in the activation of the flexor carpi ulnaris (FCU) and extensor carpi ulnaris (ECU), the absolute changes were relatively small (less than 3%). This raises the question of whether these differences are clinically meaningful. Notably, similar patterns were observed in other muscles, such as the extensor carpi radialis (ECR), where some statistically significant or trending changes (e.g., in median frequency) were accompanied by minimal percentage differences (e.g., <3%). This discrepancy may be attributed to the limited sample size and low inter-individual variability, which can enhance the sensitivity of statistical tests to small numerical differences. While these findings may reflect subtle neuromuscular adaptations, their practical implications in terms of performance enhancement or fatigue mitigation remain uncertain. Therefore, caution is warranted when interpreting the functional relevance of these changes. Future research with larger and more diverse samples, combined with objective performance outcomes, is recommended to better determine the clinical significance of these neuromuscular responses.

While this study offers important preliminary understanding of the neuromuscular adaptations related to short-term kart driving, several limitations should be considered. The relatively small sample size and the focus on adult racers competing on a single track limit the generalizability of these findings. Future research should investigate these adaptations in larger and more diverse populations, including junior drivers and those competing on various track configurations. Furthermore, electromyography (EMG) analysis of other relevant muscle groups, such as those in the upper arm and shoulder, could provide a more comprehensive understanding of the overall neuromuscular response to kart driving. Longitudinal studies examining the long-term effects of karting on muscle strength, endurance, and injury risk would also be beneficial. Given these neuromechanical complexities, future studies could benefit from integrating complementary assessment tools, such as isometric strength testing, to quantify strength adaptations in key forearm muscles, or accelerometry to capture upper-limb movement dynamics during karting. These additional metrics could help distinguish between fatigue-related changes in muscle activation and task-specific motor adaptations, providing a more comprehensive understanding of driver performance and muscular load.

## 5. Conclusions

While this study offers important preliminary understanding of the neuromuscular adaptations related to short-term kart driving, several limitations should be considered. The relatively small sample size and the focus on adult racers competing on a single track limit the generalizability of these findings. Future research should investigate these adaptations in larger and more diverse populations, including junior drivers and those competing on various track configurations. Furthermore, electromyography (EMG) analysis of other relevant muscle groups, such as those in the upper arm and shoulder, could provide a more comprehensive understanding of the overall neuromuscular response to kart driving. Longitudinal studies examining the long-term effects of karting on muscle strength, endurance, and injury risk would also be beneficial. Importantly, this study demonstrated an increase in forearm muscle activity following a short bout of kart driving, suggesting acute neuromuscular activation. However, it is plausible that longer driving sessions might lead to fatigue-induced reductions in EMG activity, which could represent a potential mechanism for overuse injuries. Incorporating this perspective into future investigations will be essential for developing effective training and injury prevention strategies tailored to kart drivers.

## Figures and Tables

**Table 1:** Electromyographic activity (normalized to maximal voluntary contraction) of forearm muscles at pre-test and post-test (n = 11).

		Pre-Test (n = 11)	Post-Test (n = 11)	Post–Pre Difference (%)	*p*-Value
ECR left	max	107.33 ± 3.55	106.53 ± 5.53	−0.74%	0.957
	mean	83.16 ± 4.40	83.93 ± 6.74	+0.93%	0.261
	median	68.13 ± 10.70	66.23 ± 24.98	−2.79%	0.18
ECR right	max	106.47 ± 7.60	107.01 ± 3.77	+0.51%	0.145
	mean	82.88 ± 5.22	84.68 ± 3.12	+2.17%	0.576
	median	85.17 ± 14.86	84.68 ± 3.12	−0.58%	0.018
ECU left	max	103.63 ± 12.29	106.30 ± 6.66	+2.57%	0.530
	mean	80.45 ± 8.32	82.07 ± 8.35	+2.01%	0.711
	median	81.11 ± 19.16	92.19 ± 21.21	+13.65%	0.119
ECU right	max	105.18 ± 6.09	107.95 ± 6.35	+2.63%	0.021
	mean	81.82 ± 5.39	83.72 ± 3.80	+2.32%	0.070
	median	87.38 ± 19.36	94.85 ± 20.18	+8.56%	0.068
FRC left	max	100.77 ± 12.42	104.70 ± 9.42	+3.91%	0.149
	mean	73.37 ± 8.80	77.74 ± 7.33	+5.96%	0.899
	median	72.97 ± 12.73	77.75 ± 7.33	+6.56%	0.081
FCR right	max	106.39 ± 6.72	107.55 ± 9.97	+1.09%	0.796
	mean	78.32 ± 7.77	79.16 ± 6.34	+1.07%	0.806
	median	81.17 ± 14.51	87.37 ± 20.01	+7.64%	0.215
FCU left	max	106.36 ± 9.81	99.79 ± 8.11	−6.18%	0.536
	mean	79.67 ± 8.91	76.53 ± 7.68	−3.94%	0.484
	median	87.09 ± 24.09	88.90 ± 18.19	+2.08%	0.498
FCU right	max	102.19 ± 7.99	104.27 ± 6.00	+2.03%	0.020
	mean	78.94 ± 6.07	78.73 ± 5.05	−0.27%	0.348
	median	101.74 ± 30.24	91.68 ± 21.00	−9.89%	0.089

ECR: extensor carpi radialis; ECU: extensor carpi ulnaris; FCR: flexor carpi radialis; FCU: flexor carpi ulnaris.

## Data Availability

The data can be provided upon a reasonable request to the corresponding author.
